# Retinal vasculitis in a patient with abdominal tuberculosis

**DOI:** 10.4103/0974-620X.64232

**Published:** 2010

**Authors:** Balaji Kannan, Kummararaj Govindarajan, Sherin Kummararaj, Vijayalakshmi Balaji, Venugopal Natarajapillai

**Affiliations:** Retina Clinic, Dr. A. Govindarajan Eye Hospital and Research Institute, Tiruchirapalli, Tamil Nadu, India

**Keywords:** Colonoscopy, C reactive protein, Eale’s disease, retinal vasculitis, serum ACE, polymerase chain reaction

## Abstract

Tuberculosis (TB) is one of the most common systemic diseases in India. Intraocular TB is however, rare. Retinal vasculitis is a relatively rare manifestion of intraocular TB. We report a case of bilateral retinal vasculitis in a 19-year-old girl with abdominal tuberculosis. The patient responded well to anti-TB treatment along with a short course of low dose oral steroids. Vision in her right eye however remained compromised due to residual maculopathy. This is the first report of bilateral retinal vasculitis due to colonic TB.

## Introduction

Tuberculosis is a chronic infection caused by *Mycobacterium tuberculosis* and *Mycobacterium bovis*. Intraocular tuberculosis is however rare (1% of all cases of TB).[1] Mycobacteria hematogenously disseminate to the eye and while the choroid is the most common site of initial involvement, TB may result in various manifestations such as scleritis, iritis, iridocyclitis, parsplanitis, choroiditis, chorioretinitis, neuroretinitis, retinal vasculitis, choroidal tuberculoma, sub retinal abscess, optic neuropathy, and endophthalmitis. TB panophthalmitis was common before the era of antiTB treatment. Retinal vasculitis, which represents small vessel inflammation involving the arterioles, capillaries, and post capillary venules, either singly or in combination, is a relatively rare manifestation.[2] We report a case of bilateral retinal vasculitis in a young patient with abdominal tuberculosis.[3]

## Case Report

A 19-year-old girl presented to our hospital with complaints of sudden diminution of vision in the right eye (OD) since morning. She gave a history of chronic abdominal pain, loss of appetite, weight loss, and chronic cough of three months duration. Gynecological history revealed amenorrhea in the last three months.

**Figure 1:**
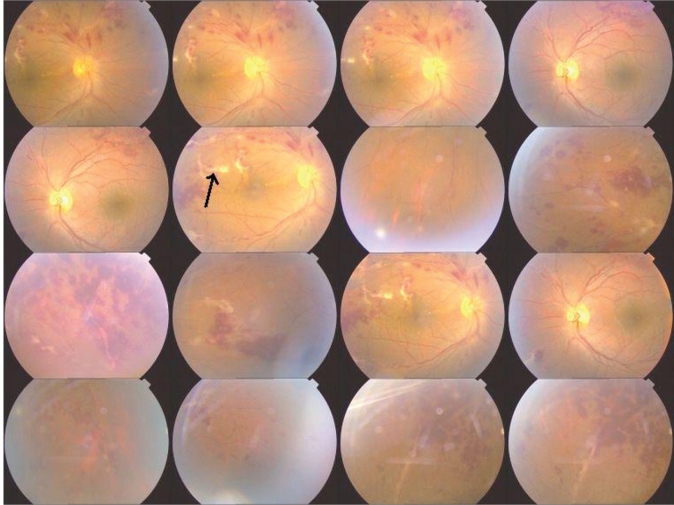
Fundus photograph showing dilated tortuous vessels with perivascular sheathing (arrow) with scattered areas of superficial and deep retinal hemorrhages along the superior, nasal and temporal quadrants with macular edema OD and scattered hemorrhages in all the quadrants OS. There was no macular involvement OS

**Figures 2-4:**
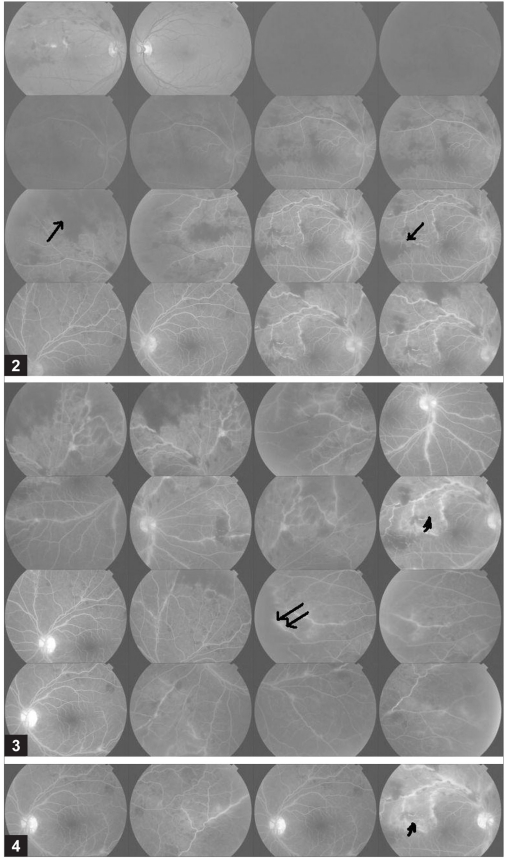
Fundus fluorescein angiogram showing extensive areas of hypo fluorescence mainly due to retinal hemorrhages causing blocked fluorescence (arrow) and disc staining in the late phase OU. Some leakage of the dye from the capillaries (arrow head) in the affected segment is seen OD. A small zone of capillary non perfusion (double arrow) is seen in the nasal quadrant OS

On examination, vision OD was counting fingers at five meters and 20/40 in the left eye (OS). Extra ocular movements were full. Anterior segment evaluation with slit lamp biomicroscope was normal. There were few anterior vitreous cells in both eyes (OU). Fundus examination (OD) media clear, disc (size, shape and color) normal, macular edema superiorly and dilated tortuous (superotemporal and inferotemporal tributary veins) with perivascular sheathing and scattered areas of superficial and deep retinal hemorrhages in midperiphery of all the quadrants. In OS media clear, disc normal, macula normal, tortuous superotemporal tributary veins with hemorrhages in midperiphery of all the quadrants [Figure 1]. Fundus fluorescein angiography showed extensive areas of hypo fluorescence (blocked fluorescence due to retinal hemorrhages) and disc staining in the late phase OU. Some leakage of the dye from the capillaries in the affected segment is seen OD. A small zone of capillary non perfusion area noted in the nasal quadrant of OS [Figures 2–4].

Hemoglobin was 8.5mg/dl and peripheral blood smear showed microcytic hypochromic red blood cells. ESR was found to be 45 mm at 1^st^ hour which are performed routinely in our hospital for patients with retinal vasculitis. Serological tests namely rheumatoid arthritis factor and antinuclear antibody were negative. C-reactive protein (CRP) and serum angiotensin converting enzyme (SACE) levels were within normal range. Stool test for occult blood was negative. Chest X-ray and barium meal study were normal. Ultrasound abdomen showed free fluid in the cul de sac and retro peritoneum. Mantoux test was positive at 13×13 mm. The patient underwent colonoscopy by surgical gastroenterologist, which showed ulcers in the ascending colon and was diagnosed as colonic TB on the basis of clinical appearance and histopathological evidence.

A diagnosis of retinal vasculitis secondary to colonic TB was made and the patient was commenced on antiTB therapy Tab. Isoniazid 300mg, Tab. Rifampicin 600mg, Tab. Ethambutol 800mg and Tab. Pyrazinamide 1000mg along with a short course of low dose oral steroids (oral prednisolone 1mg/kg started with 40mg/day and tapered 10mg/week for 4 weeks.)

**Figure 5:**
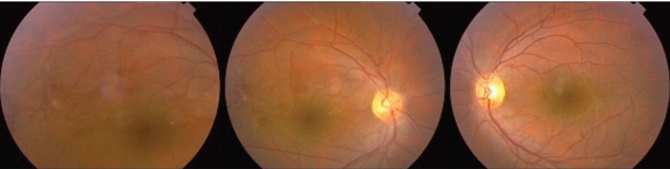
Fundus photograph OU showing resolution of vasculitis and hemorrhage after three months of treatment

**Figures 6,7:**
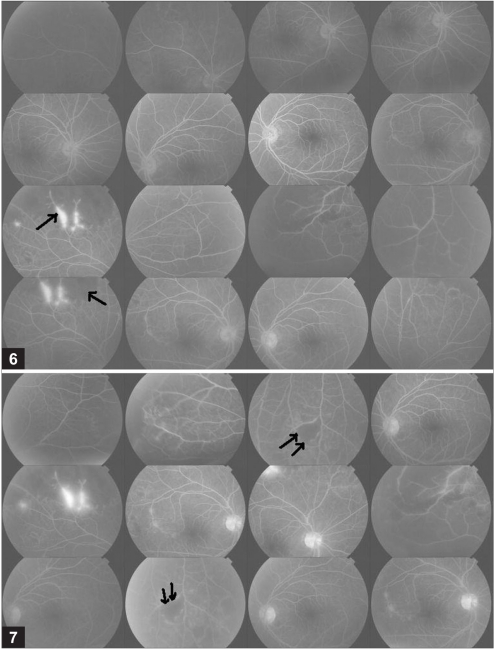
Fundus fluorescein angiogram showing staining of venules with adjoining capillary non perfusion (arrow) OD and areas of areas capillary non perfusion OS in the left eye (double arrow)

**Figure 8:**
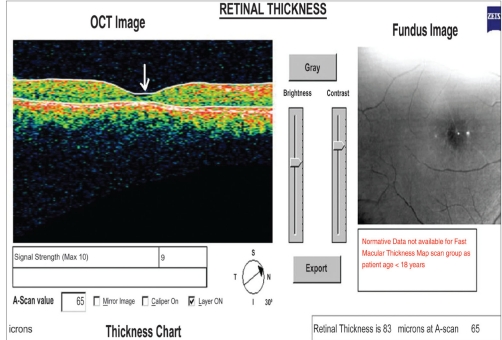
Optical coherence tomogram showing foveal thinning (arrow) OD

She was monitored every month and three months after treatment, fundus examination OU showed resolution of retinal vasculitis [Figure 5]. Fundus fluorescein angiography showed resolution of hemorrhages, staining of venules OD with adjoining CNP [Figures 6 and 7]. Sectoral scatter photocoagulation of areas of capillary dropouts was performed OU. At the last follow up, her best corrected visual acuity OD was 20/120 and 20/20 OS. Optical Coherence Tomography (OCT) showed foveal thinning (83microns) OD and normal foveal thickness (143 microns) OS [Figure 8].

## Discussion

The large variations in clinical presentation and the lack of uniformity in diagnostic criteria make the diagnosis of intraocular TB difficult. In most cases diagnosis requires corroborative evidence, such as a positive PPD and chest x-ray, or a positive therapeutic trial for TB with exclusion of other causes (presumed ocular TB). Direct evidence for the presence of the infectious agent in the eye by demonstration of acid-fast organisms under the microscope or the detection of bacterial genome by polymerase chain reaction (PCR) is required for confirmation (confirmed ocular TB).[4]

There are several reports of retinal vasculitis due to TB. Gupta and co workers described thirteen patients of retinal vasculitis.[4] In all patients PCR of intraocular fluid (aqueous and vitreous) was positive for Mycobacterium TB. Rosen and co workers described a series of twelve patients with intraocular TB; nine of them had florid ischemic retinal vasculitis.[5] Hoh *et al*. reported a case of bilateral retinal periphlebitis which responded promptly to two months of antiTB treatment.[6] Eale’s disease is a form of retinal vasculitis predominantly affecting the peripheral retina of young and otherwise healthy adults between 15-40 years. Biswas *et al*. demonstrated Mycobacterium TB by nested PCR supporting the association of Mycobacterium TB in Eale’s disease.[7]

The differential diagnosis in our case included collagen vascular disease, sarcoidosis and hematological disorder; however these were ruled out by investigations. Antitubercular therapy in a patient of suspected tubercular retinal vasculitis should be four drug regimens which include isoniazid 5 mg/kg/day, rifampicin 450 mg daily if body weight is less than 50 kg and 600 mg/day if body weight is more than 50 kg, ethambutol 15 mg/kg/day and pyrazinamide 20 mg/kd/day for first four months followed by rifampicin and isoniazid for 9-14 months.

Our case is the first report of bilateral retinal vasculitis due to colonic TB. Thorough systemic evaluation in patients with retinal vasculitis to detect TB and prompt treatment with antiTB therapy can preserve vision. Prompt diagnosis and treatment with antiTB therapy led to an improvement in our patient. Vision in her right eye however remained compromised due to residual maculopathy.
